# Efficacy and Safety of Nivolumab for Advanced Renal Cell Carcinoma: A Systematic Review and Meta-Analysis

**DOI:** 10.1155/2022/5430525

**Published:** 2022-03-25

**Authors:** Siqi Zhang, Xiaohua Xu, Jiaqi Chen, Zhiping Zhang, Feng Liu

**Affiliations:** Department of Nephrology, China-Japan Union Hospital of Jilin University, Changchun, China

## Abstract

**Objective:**

To assess the efficacy and safety of nivolumab for advanced renal cell carcinoma (RCC) via meta-analysis.

**Methods:**

In this systematic review and meta-analysis, we searched PubMed, Embase, Science Citation Index Expanded, The Cochrane Library, and Web of Science for randomized controlled trials (RCTs) using nivolumab for patients with advanced RCC published before 30 December 2021. Quality assessments and meta-analyses were performed on all the literature assessed for eligibility.

**Results:**

Of 203 studies identified as potentially eligible from 3214 studies in a preliminary search, three RCTs including 2550 RCC cases met the inclusion criteria and were of high quality. Meta-analysis showed benefits of nivolumab in the progression-free survival (PFS) (HR = 0.73, 95% CI: 0.54 to 0.99, *P*=0.04) and overall survival (OS) (HR = 0.70, 95% CI: 0.63 to 0.78, *P* < 0.001) of patients with advanced RCC, and no increase in documented adverse events was recorded.

**Conclusion:**

Nivolumab plus ipilimumab has significant benefits versus sunitinib in the treatment of advanced RCC in terms of tumor progression control and prolongation of OS and PFS, with a manageable safety profile.

## 1. Introduction

Renal cell carcinoma (RCC) is a common pathological type of renal tumor, accounting for 80% to 90% of all renal cancer [1]. The lack of specific clinical signs in early RCC results in the diagnosis of clinical stage T3 in approximately 60% of patients, with metastases found in some cases [2]. Surgery is currently the treatment of choice for locally advanced RCC. However, advanced RCC also requires chemotherapy or radiotherapy in addition to palliative treatment with radical nephrectomy or cytoreductive nephrectomy [3]. Targeted drug therapy, a conventional approach for the treatment of malignant tumors, provides a satisfactory clinical efficacy and substantial prolongation of overall survival (OS), among which sunitinib is a novel multitargeted oral drug for RCC. Research has demonstrated the excellent efficiency of sunitinib in the treatment of advanced RCC with manageable safety and tolerability [4].

In recent years, with the advancement of tumor immunology research, specific targeted immunotherapy based on programmed death-1 (PD-1) and programmed death molecular ligand-1 (PD-L1) inhibitors are increasingly used for various malignancies [5, 6]. Nivolumab, a representative PD-1 inhibitor, was the first drug adopted for the treatment of advanced squamous non-small cell lung cancer. Choueiri et al. [7] applied nivolumab for metastatic RCC and found its immunomodulatory role in effectively enhancing T-cell responses and cytokine production. Ipilimumab is the first cytotoxic T lymphocyte-associated protein 4 (CTLA-4) immune checkpoint inhibitor that increases T cell activation and proliferation by blocking CTLA-4 binding to its ligand (CD80/CD86) and participates in the tumor immune response. Currently, ipilimumab is only approved for the treatment of metastatic melanoma and postoperative adjuvant therapy for melanoma patients, so it is more often used clinically in combination with PD-1 inhibitors such as nivolumab for advanced solid tumors. The International Metastatic Renal Cell Carcinoma Database Consortium (IMDC) has also recommended nivolumab plus ipilimumab as the standard first-line treatment for RCC. Nivolumab plus ipilimumab has been reported to be associated with improved prognosis of RCC patients [8]. Here, we did an electronic search on English databases to perform a meta-analysis of RCTs using nivolumab, ipilimumab, and sunitinib for advanced RCC, to provide more evidence for the clinical treatment of advanced RCC.

## 2. Materials and Methods

### 2.1. Search Strategies

The literature search strategy was developed as per the relevant requirements of the Cochrane Handbook for Systematic Evaluators. The English and Chinese literature search terms were “renal carcinoma,” “renal cell carcinoma,” “kidney tumor,” “advanced stage,” “metastatic,” “treatment,” “Opdivo/nivolumab,” “Yervoy/ipilimumab,” “sunitinib,” and “immunotherapy.” The search was carried out in the English databases (PubMed, Embase, Science Citation Index Expanded, The Cochrane Library, and Web of Science). The publication date of the searched literature was before 30 December 2021. References of the included literature were searched and retrospectively added to potentially missing studies whenever possible. Two investigators independently assessed the studies for inclusion and evaluated their quality as per the Jadad scoring criteria and the Cochrane Handbook for Systematic Evaluators. Extracted data mainly included study publication information, baseline features of study subjects, and their comparability, interventions, and clinical efficacy, using independent extraction and cross-checking.

### 2.2. Inclusion and Exclusion Criteria

#### 2.2.1. Inclusion Criteria

(1) Study types included global RCTs on nivolumab for advanced RCC, regardless of allocation concealment or masking. The language of the literature was limited to Chinese and English. (2) Study subjects were aged 18 to 80 years and diagnosed with advanced RCC by pathological biopsy [9]; with evaluable lesions; without other antitumor treatment before randomization; with normal liver and kidney function; and without hematologic and cardiovascular diseases [10, 11]. (3) Interventions: the trial group was given nivolumab monotherapy or combination therapy, and the control group was given other treatments. (4) Primary endpoints: one or more of OS, progression-free survival (PFS), and adverse events (AEs) were included.

#### 2.2.2. Exclusion Criteria

(1) Prospective nonrandomized controlled trials, retrospective studies, or case reports; (2) Animal experiments and in vitro experiments; (3) Literature with multiple publications or subgroup analysis; (4) Literature in the form of reviews, experimental plans, or expert reviews.

### 2.3. Quality Assessment of Studies

Assessment criteria are as follows: (1) whether the randomization was an RCT, (2) whether the allocation was concealed, (3) whether the study subjects, study protocol operators, and study results adjudicators were blinded, and (4) whether there were patients with the withdrawal of consent or loss to follow-up and whether intentional analysis was conducted. The quality assessment of the literature was trichotomized to level A, level B, and level C. Level A is “correct or adequate” for all assessment criteria, with a low probability of biases. Level B is one or more indicators that are poorly described or partially satisfied, with a medium probability of biases. Level C is any one or more indicators not satisfied or absent, with a high probability of biases. In case of discordance between the two investigators during the assessment, a third investigator was consulted to resolve the quires, and the predesigned form was used to extract the baseline data, interventions, and endpoints of the study subjects from the literature, which were collated by the fourth investigator.

### 2.4. Statistical Analysis

Meta-analysis was performed using the Review Manager 5.2 software for a comprehensive quantitative analysis of the literature results. Survival data such as PFS and OS were expressed as the hazard ratio (HR) and its 95% confidence interval (CI), and differences were considered statistically significant at *P* < 0.05. Statistical heterogeneity among the included studies was analyzed using the chi-square test, while the I2 test was used for quantitative analysis of heterogeneity with a test level of *α* = 0.1. (1) When *P* ≥ 0.1 and I2 ≤ 50%, the heterogeneity among the included studies was considered not statistically significant, and meta-analysis was performed using a fixed-effects model. (2) When *P* < 0.1 and I2 > 50%, the heterogeneity among the included studies was considered statistically significant, and if there was still no significant clinical heterogeneity after further analysis, meta-analysis was performed using a random-effects model, and vice versa for descriptive analysis.

## 3. Results

### 3.1. Literature Search Results

A total of 203 articles were considered potentially eligible among the 3214 articles in the search results, and three RCTs were assessed for eligibility for inclusion [12–14], involving 2550 cases of advanced RCC. Differences in efficacy were compared between nivolumab plus cabozantinib versus sunitinib, nivolumab plus ipilimumab versus sunitinib, and nivolumab versus everolimus, respectively. The basic characteristics of the eligible literature are shown in Table 1.

### 3.2. Quality Assessment of the Eligible Literature

The eligible studies were all multicenter RCTs, with good consistency and comparability of baseline features between the patients in different groups in the studies. The quality of the papers was high with two articles in level A and one in level B. The integrity of the reported results is promising and no information deficiency biases were found (see Table 2).

### 3.3. PFS and OS Analysis

**Figure 1:**
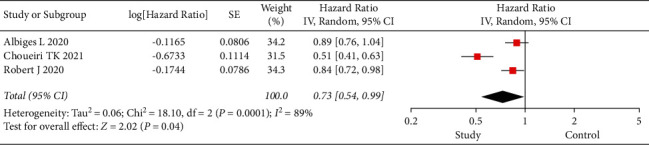
Forest plot of PFS. PFS, progression-free survival; CI, confidence interval.

**Figure 2:**
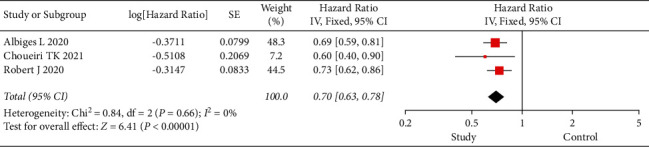
Forest plot of OS. OS, overall survival; CI, confidence interval.

The PFS and OS of subjects were investigated in the three eligible studies (Figure 1). In the analysis of PFS in all subjects, there was significant within-group heterogeneity (I2 = 89%, *P* < 0.001), and the results via the random-effects model showed an HR of 0.73 and 95% CI of 0.54 to 0.99 (*P*=0.04). In the analysis of OS in all subjects, there was no within-group heterogeneity (I2 = 0, *P*=0.66), and the results of the fixed-effects model showed an HR of 0.70 and 95% CI of 0.63 to 0.78 (*P* < 0.001) (Figure 2). Nivolumab showed PFS and OS benefits in patients with advanced RCC.

### 3.4. AEs Analysis

**Figure 3:**
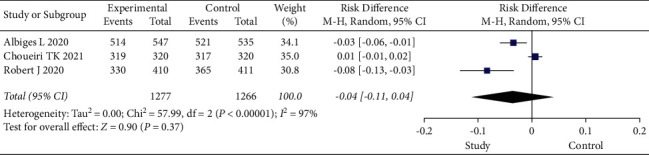
Forest plot of AEs. AEs, adverse events; CI, confidence interval.

Adverse events (AEs) (nausea and vomiting, diarrhea, increased lipase, increased amylase, increased alanine aminotransferase, hypertension, and malaise) during treatment were analyzed in the three eligible studies. In the analysis of AEs in all subjects, there was significant within-group heterogeneity (I2 = 97%, *P* < 0.001), and the results of the random-effects model showed a risk difference (RD) of −0.04 and 95% CI of −0.11 to 0.04 (*P*=0.37), indicating that nivolumab had a manageable safety profile (Figure 3).

## 4. Discussion

To provide a more favorable basis for decision-making on clinical treatment regimens for patients with advanced RCC, this article evaluated the efficacy and safety of nivolumab in the treatment of advanced RCC via meta-analysis. Meta-analysis in this article showed a significant prolongation of PFS (HR = 0.73, 95% CI: 0.54 to 0.99, *P*=0.04) and OS (HR = 0.70, 95% CI: 0.63 to 0.78, *P* < 0.001) in patients with advanced RCC by nivolumab, suggesting that for patients with advanced RCC, nivolumab may provide an optimal alternative to significantly alleviating patients' symptoms and prolong survival. Most of the AEs associated with nivolumab, ipilimumab, and sunitinib are grade I∼II, which have little impact on the patient's daily life and can be relieved without medical intervention. However, the AEs of sunitinib were mainly hypertension, which requires close monitoring of blood pressure changes during treatment and some auxiliary methods (such as weight control, low-salt diet, and appropriate regular exercise) to help blood pressure control. Since nausea, vomiting, and fatigue are the main AEs of nivolumab plus ipilimumab, attention should be attached to the gastrointestinal reactions of patients during treatment and appropriate nutritional supply. In general, the meta-analysis showed that the incidence of AEs of nivolumab plus ipilimumab was higher than that of sunitinib, but the difference was not statistically significant, so the safety of nivolumab is considered manageable.

Patients with RCC usually show a promising prognosis after surgical treatment, but postoperative recurrence and metastasis were still found in 30% to 50% of patients [15]. Due to the insidious early clinical symptoms of RCC, approximately 30% to 40% of patients experience metastases at the time of diagnosis, known as an advanced stage or metastatic RCC [16]. Patients with advanced RCC are unresponsive to conventional radiotherapy and chemotherapy, and even immunotherapy based on interferon and interleukin is marginally effective [17]. In recent years, targeted therapy has been considered one of the most effective treatment modalities for patients with advanced, especially metastatic RCC. Sunitinib, a dual-channel, multitarget tyrosine kinase inhibitor, is one of the most-used targeted therapeutic agents at present. Pharmacological experiments have confirmed that sunitinib inhibits the growth of cancer cells, blocks the blood and nutrient supply required for tumor growth, and has been widely used for many solid tumors, including RCC [18]. However, the use of sunitinib is associated with a high incidence of AEs, despite its high effectiveness [19]. RCC is a highly immunogenic malignancy with significant upregulation of PD-1 or PD-L1 expression in about 30% of RCC tissues. The only immunologic agent currently approved for the treatment of metastatic RCC is nivolumab, a PD-1 inhibitor. A study suggested that nivolumab offers a new treatment alternative for patients with advanced RCC [20]. Motzer et al. [21] reported that in previously treated RCC patients, the use of nivolumab was effective in improving overall efficiency and objective remission rates and prolonging median survival with a high safety profile, with only 2% of patients experiencing fatigue during treatment. Ipilimumab was initially used in the treatment of metastatic melanoma, and data [22] showed a good survival benefit and a durable and complete response in some patients with refractory advanced disease. Since nivolumab is a PD-1 immune checkpoint inhibitor and ipilimumab is an immune drug targeting the T-cell CTLA-4 protein, the combination of the two has received widespread attention for the treatment of advanced tumors. It has been reported [23] that nivolumab plus ipilimumab provides a manageable safety profile and shows rapid and profound tumor regression in a significant proportion of patients. However, there were still some shortcomings in this meta-analysis. At present, there are many kinds of targeted drugs for RCC. In this meta-analysis, the number of included studies was small and the interventions varied from studies. In addition, we just enrolled studies of nivolumab, with no comparison with other drugs.

## 5. Conclusion

Nivolumab plus ipilimumab have significant benefits versus sunitinib in the treatment of advanced RCC in terms of effective control of tumor progression and prolongation of patient's OS and PFS, with a manageable safety profile. The limitation of this study is the small number of the included literature. We look forward to more large-sample, high-quality RCTs on related studies in the future to further clarify the efficacy and safety and provide a more reliable basis for the treatment of advanced RCC.

## Figures and Tables

**Table 1:** Basic characteristics of the eligible literature.

Author	Year	Registered name	Phase	No.	Arm 1	Arm 2	Endpoints
Choueiri et al. [12]	2021	CheckMate 9ER	III	651	Nivolumab plus cabozantinib	Sunitinib	PFS, OS, AEs
Motzer et al. [13]	2020	CheckMate 025	III	803	Nivolumab	Everolimus	PFS, OS, AEs
Albiges et al. [14]	2020	CheckMate 214	III	1096	Nivolumab plus ipilimumab	Sunitinib	PFS, OS, AEs

PFS, progression-free survival; OS, overall survival; AEs, adverse events.

**Table 2:** Quality assessment of the eligible literature.

Eligible study	Randomization	Allocation concealment	Blinding	Withdrawal	ITT analysis	Baseline features	Quality level
Choueiri et al.	Stratified randomization	Unclear	Open-label	Yes	Yes	Similar	B
Motzer et al.	Stratified randomization	Yes	Open-label	Yes	Yes	Similar	A
Albiges et al.	Stratified randomization	Yes	Open-label	Yes	Yes	Similar	A

ITT, intention-to-treat.

## Data Availability

The datasets used during the present study are available from the corresponding author upon reasonable request.
